# Some American Hospitals

**Published:** 1892-03-05

**Authors:** 


### Some American Hospitals.

By Our Special Commissioner.

### X.—THE MASSACHUSETTS GENERAL HOSPITAL, BOSTON.

This noble institution, one of the oldest and best in the States, still maintains its position for efficiency and good work. Since we visited it in 1882, poor Dr. Whittemore, whose terrible sufferings won for him the sympathy of everybody, has passed away. The memory of his kindness and hospitality was with us during the whole of our visit to Boston, and we could not fail to miss his kindly face, or to regret the absence of a courteous presence familiar with all matters connected with the welfare and progress of American hospitals. Dr. Pratt, the present Superintendent, is a graduate of the hospital school, and has its interests fully at heart. He gladly showed us all the new features of the hospital, some of which are especially valuable. Three thousand five hundred and seventy-two patients were admitted during the year, of whom 2,719 were free, and the rest paid wholly or in part for their maintenance. The greatest number occupying beds throughout the year was 225, the average number of patients being 198. It is a curious fact that the paying patients found it desirable or necessary to reside in the hospital on an average for 3·55 weeks each. Some idea of the amount contributed by pay patients will be gathered from the fact that the highest amount paid by any one patient was 70 dols., and the lowest 4·00 dols. The maximum sum charged to pay patients is 50 dols. per week; in smaller rooms, 25· dols. per week; in the general ward, 10· dols. per week. These rates only covered the expenses of board and lodging. The hospital maintains an ambulance, which is despatched, together with a medical officer, to any point within the city proper for the convenience of cases of accident, or urgent sudden sickness not contagious. At this hospital, a notice from a physician, the police, or other responsible person, entitles the poor to early gratuitous admission.

The new features of this hospital since our last visit are the gynæcoligical ward and theatre, and Kingley's photographic studio. The former is so constructed as to be entirely isolated, and the strictest aseptic precautions are taken. Otherwise, the building proper offers no specially novel features of construction, except its theatre. This operation theatre contains the largest window, and has undoubtedly the very best light of any theatre we have ever seen. It is of a convenient size, and well constructed in every way. Visitors and students approach it by a separate staircase from outside, so that no one is allowed to enter the wards except the physicians and nurses in charge. One drawback to the theatre seemed to us to be that it was completely raked by the windows in the Nurses' Home. The effect of this is minimised by the blinds, but on dark days especially the situation must prove objectionable on this account. Kingsley's photographic studio has been fitted up by one of the students who was much interested in the subject, and who, poor fellow, lost his life from diphtheria a short time ago. Here all the most interesting cases and specimens are photographed, an arrangement which materially aids the staff in their clinical work. All hospitals attached to medical schools should certainly possess a photographic studio.

We are glad to see that the strictest attention is paid to cleanliness throughout this hospital, and that Dr. Pratt is evidently determined to maintain efficiency in all departments, and it is fair to conclude that he is ably seconded by the officers who have immediate charge of them. The expenditure of the hospital amounted to $120,000 in round numbers, the income being only $116,000, leaving a deficiency of $4,000. This is a decided improvement on the financial condition of the previous year, 1889, when the deficiency amounted to nearly $26,000. This is one of the few hospitals in the United States which have convalescent homes attached to them. The home is excellently situated, and receives inmates from the other Boston hospitals and institutions. Altogether 386 patients were under treatment during the year.

#### The Nursing Department.

It is a remarkable fact, but it is a fact, that the seventy-seventh annual report of the trustees of the Massachusetts General Hospital and McLean Asylum of 1890 contains no reference whatever to the nursing department. Dr. Cowles, whose indomitable energy is notorious, has given ten pages of excellent stuff concerning the McLean Asylum Training School for Nurses, but of the hospital nurses not one word is mentioned. This is a strange omission for which we are unable to account, seeing that the nursing school of the hospital is one of the oldest and most important of the United States. Nurses are often made too much fuss of, but to ignore them altogether in the annual report is so unique as to call for some explanation. The condition of the hospital to-day shows that the nurses discharge their duties in an excellent manner. They are provided for in a special home erected in 1885, and each nurse has a room entirely to herself. It is part of the regulations of the hospital that every nurse, when off duty, shall leave the hospital buildings and either take exercise or return to the Home. Here sitting and reception rooms are provided, which are comfortably furnished, the pictures being especially good. The nurses have been provided with the largest and best selected library we have seen anywhere in connection with a training school for nurses. If a probationer at the Massachusetts General Hospital employs her leisure, she may acquire a sound knowledge of many things by reading the books contained in the nurses' library here. The whole of the meals are served in the rotunda dining-room, which used to be the old operating theatre. It is centrally situated, so that the nurses can reach it from every part of the hospital without difficulty, and has been so well adapted to its purpose that it makes the finest nurses' dining-room to be found in any hospital. All the arrangements for the nurses' comforts are good, and we should imagine that the staff must find themselves both comfortable and healthy at this institution. Miss Myra B. Brown is Superintendent of nurses, but we, unfortunately, did not have the pleasure of making her acquaintance, as she was out at the time of our visit. Although the rules of most of the nurses' training school provide for the sending out of nurses to private cases, in practice very few institutions attempt this work, and where it is attempted, only a few nurses are employed. The American public send to the registry of nurses, which is usually connected with the College of Physicians and Surgeons. The nurses pay a fee of five dollars on registration, and the Committee reserve the right to remove the name of any nurse from the registry at their discretion. The Committee decline to make any terms of employment for nurses. It only undertakes to send nurses as applicants for employment, leaving them to make their own terms and arrangements with employers.

The first directory for nurses was established at Boston, Mass., in November, 1879, and opened with the names of sixty nurses on its books. The chief members of the medical profession in practice around Boston were communicated with, askingt hem to supply a list of such nurses, as from personal knowledge, they could recommend. To all nurses so vouched for, as well as to all graduates of the training schools for nurses, circulars were then sent explaining the plan, and notifying them to register themselves. At first a nominal fee of fifty cents for registration was charged, which has now been increased to five dollars. We have had to explain the details of the working of this system in the "Nursing Mirror Supplement," in connection with the nurses' co-operation, on many occasions, and we need not, therefore, repeat them here. The chief aim of the directory is to put employers and nurses in direct communication with each other, and to inform as to the character of one who is about to become an important member of the household. A book is kept containing the name and details of each nurse's training and experience. Into this book are entered every engagement which the nurse has had through the directory, the report which was received from the attending physician, and also from the patients, as to the conduct of the nurse in every case. Should either or both of these reports be unsatisfactory, a general committee of ladies and gentlemen representing the directory investigate the charges made and on their finding the Committee either exonerate the nurse or award her one of three penalties—(1) a caution, (2) a fine of three dollars, or (3) the removal of her name from the list. No advertisements have appeared in the papers of this directory, which relies upon its merits solely for its success. No difficulty whatever is experienced in furnishing full employment to all really desirable nurses, the demand keeping pace with the supply. The directory of Boston owes its establishment and success to two doctors, viz., F. C. Shattuck and C. P. Putnam. It is the first of its kind ever established, and has led to the formation of similar ones in Philadelphia, New York, and other large cities. The fees paid by the public to the directory of nurses amounted in the following years to 2,253 dols. in 1889, 2,602 dols. in 1890, and 2,925 dols. in 1891. This shows a steady progressive increase, which is likely to continue, we understand, thanks, in no small degree, to the intelligent care and devotion of Dr. Edward H. Brigham, the Registrar. At the present time there are about one thousand nurses on the registry at Boston. It may be added that the fee charged the public for furnishing the addresses of nurses from 8 a.m. to 8 p.m. is 1 dol., and from 8 p.m. to 8 a.m. 2 dols. An extra charge is made in cases requiring unusual labour and responsibility —as when nurses are found and sent in answer to applications, by letter, telegram, or telephone.

